# Rapid identification of primary atopic disorders (PAD) by a clinical landmark-guided, upfront use of genomic sequencing

**DOI:** 10.5414/ALX02520E

**Published:** 2024-10-02

**Authors:** Tim Niehues, Sandra von Hardenberg, Eunike Velleuer

**Affiliations:** 1 Center for Child and Adolescent Health, Helios Hospital Krefeld, Academic Hospital of RWTH Aachen, Krefeld,; 2 Department of Human Genetics, Hannover Medical School, Hannover, and; 3 Department of Cytopathology, Institute of Pathology, Heinrich Heine University Düsseldorf, Germany

**Keywords:** primary atopic disease, inborn error of immunity, whole genome sequencing, rapid diagnosis

## Abstract

Primary atopic disorders (PAD) are monogenic disorders caused by pathogenic gene variants encoding proteins that are key for the maintenance of a healthy skin barrier and a well-functioning immune system. Physicians face the challenge to find single, extremely rare PAD patients/families among the millions of individuals with common allergic diseases. We describe case scenarios with signature PAD. We review the literature and deduct specific clinical red flags for PAD detection. They include a positive family history and/or signs of pathological susceptibility to infections, immunodysregulation, or syndromic disease. Results of conventional laboratory and most immunological lab studies are not sufficient to make a definitive diagnosis of PAD. In the past, multistep narrowing of differential diagnoses by various immunological and other laboratory tests led to testing of single genes or gene panel analyses, which was a time-consuming and often unsuccessful approach. The implementation of whole-genomic analyses in the routine diagnostics has led to a paradigm shift. Upfront genome-wide analysis by whole genome sequencing (WGS) will shorten the time to diagnosis, save patients from unnecessary investigations, and reduce morbidity and mortality. We propose a rational, clinical landmark-based approach for deciding which cases pass the filter for carrying out early WGS. WGS result interpretation requires a great deal of caution regarding the causal relationship of variants in PAD phenotypes and absence of proof by adequate functional tests. In case of negative WGS results, a re-iteration attitude with re-analyses of the data (using the latest data base annotation)) may eventually lead to PAD diagnosis. PAD, like many other rare genetic diseases, will only be successfully managed, if physicians from different clinical specialties and geneticists interact regularly in multidisciplinary conferences.

## Introduction 

### Primary atopic disorders (PAD) – single drops in the ocean of allergies 

Atopy (from Greek “strangeness”) is defined as “a personal, and/or familial tendency to produce IgE antibodies in response to ordinary exposure to allergens, usually proteins.” Allergies manifest via the interaction of environmental factors (e.g., diet, microbes, pollution, etc.) and host immunity. Inborn errors of immunity (IEI) are > 500 disorders in which parts of the immune system are missing, defective or dysfunctional due to monogenic germline variants that result in loss of function (LOF), gain of function (GOF), or multimorphic altered function of the encoded protein. The term PAD refers to a subgroup of IEI with a phenotype that includes allergic diseases as well as genetic disorders of the skin barrier. 

Allergic diseases are extremely common (estimated to affect up to 20 – 30% of the world’s population), while PAD are extremely rare. Therefore, this makes the timely detection of PAD individuals in the large sea of allergic individuals a big challenge. A delay in the diagnosis of PAD is associated with a significant increase in morbidity and mortality. Additionally, for affected patients and families, the diagnostic odysseys across many specialties with frequent, sometimes quite invasive investigations, is a huge burden. Therefore, we propose a strategy to identify PAD more rapidly and reliably to reduce morbidity and mortality for the affected individuals. 

## Part 1

### Sailing the seven seas: Typical PAD case scenarios grouped by the major pathophysiological traits

This review covers the description of the molecular immunology and physiology of skin barrier (keratinocytes), innate immunity (granulocytes, mast cells), and adaptive immunity cells (T cells, regulatory T cells (Tregs)). Others and us group PAD by major pathophysiological mechanisms [1, 2, 3, 4, 5, 6, 7, 8]. We define clinical landmarks (red flags) and describe the atopic and clinical presentation. 

## I. Disorders affecting the skin barrier function 

**RED FLAG: Newborns or infants with failure to thrive, generalized ichthyosiform erythroderma, and hair anomalies (e.g., bamboo hair), diarrhea as signs for disrupted skin epithelial and mucosal barrier function.**

### SIGNATURE DISEASE: Netherton syndrome 

biallelic LOF variants in *SPINK5*

**Pathophysiology**

Increased skin permeability allows entry of allergens and other foreign antigens (toxins, e.g., *Staphylococcus aureus* toxin) that aggravate inflammation. Moreover, damaged keratinocytes release alarmins such as thymic stromal lymphopoietin (TSLP), IL33, and IL25 that promote a type 2 inflammatory response (IgE production, skewing towards T helper 2 cells (TH2)/IL4/IL5, IL13, and IL31 and activation of type 2 innate lymphoid cells (ILC2s)). 

**Clinical signs suggesting allergy**

Common complications are recurrent anaphylactic episodes mediated by specific IgE antibodies leading to urticarial rashes, facial angioedema, and often extracutaneous reactions, triggered by certain foods. Food allergy usually manifests in infancy and early childhood. 

**Other clinical signs**

Erythroderma is found directly at or a few days after birth accompanied by ichthyosis that may present as serpiginous erythema with double-edged scales (ichthyosis linearis circumflexa). Pruritus is usually severe and has an extreme influence on disease burden. Affected patients develop multifactorial failure to thrive mainly due to high energy loss through skin inflammation and hyperproliferation (“dermopathic enteropathy”). Newborns are prone to hypernatremic dehydration, severe skin infections, and systemic sepsis. Growth hormone deficiency may occur in some affected individuals and is often unrecognized. The impaired epidermal barrier is associated with risk of systemic toxicity from topically applied agents. Children usually display hypotrichosis with thin, spiky, and fragile hair of slow growth (trichorrhexis invaginata (“bamboo hair”)). 

**Figure 1. Figure1:**
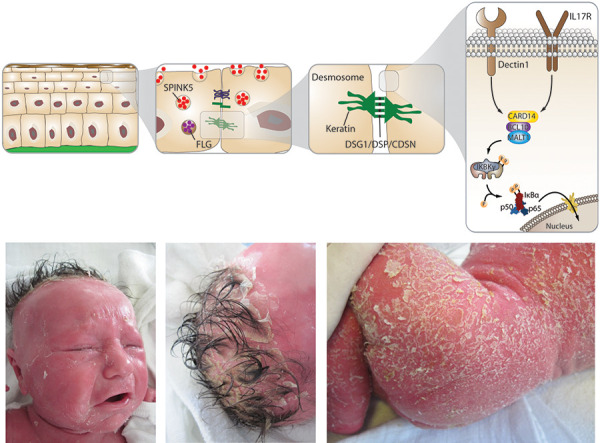
Physiology of the normal skin/mucosa barrier (upper panel) and newborn of 37 + 6 weeks gestation with Netherton Syndrome (pathogenic variants in *SPINK5*): Ichthyosiform erythroderma with generalized scaling, severe cutaneous inflammation resulting in debilitating pruritus and extremely sensitive skin (lower panel, provided by Dr. Arpe, St. Marien Hospital, Düren, Germany). *SPINK5* (serine protease inhibitor Kazal-type 5) encodes the multidomain serine protease inhibitor LEKTI (lymphoepithelial Kazal-type-related inhibitor), which controls the kallikrein network of the epidermis and regulates desmosome turnover. Desmosomes seal the keratinocyte network. Corneodesmosin (CDSN), Desmoglein1 (DSG1), or Desmoplakin (DSP) are key intercellular adhesion molecules. Filaggrin monomers contribute to epidermal water retention through their hygroscopic properties. Activation of Dectin 1 or IL17 receptor (IL17R) leads to activation of CARD14 and NFκB, important for keratinocyte homeostasis (upper panel from left to right).

Other PAD with disrupted epithelial and mucosal skin barrier are presented in Table 1 [9, 10, 11]. [Fig Figure1]

## II. Disorders affecting the innate immune system: granulocytes, mast cells 

**RED FLAG: At any age, families with vibratory (e.g., jogging) and/or cold-induced (e.g., swimming pool)) urticarial rashes non-responsive to antihistamines with a peculiar history (e.g., periodic fever) as signs for dysregulated granulocyte and/or mast cell function.**

### SIGNATURE DISEASE: PLAID (PLCγ2-associated antibody deficiency and immune dysregulation or familial cold autoinflammatory syndrome 3 (FCAS3)) 

heterozygous GOF/LOF variants in *PLCG2* autosomal dominant (AD) 

**Pathophysiology**

GOF variants in *PLCG2* lead to an elevated basal activity of PLCγ2 and the production of IP3 and diacylglycerol (DAG) (Figure 2A, left panel). The temperature sensitivity in PLAID is characteristic. Mast cells are spontaneously activated following exposure to temperatures lower than 37 °C, which explains the occurrence of cold urticaria. Hypogammaglobulinemia is a prominent feature of the disease, with decreased numbers of class-switched memory B cells, and circulating CD19+ B cells and NK cells [8]. 

**Clinical signs suggesting allergy**

PLAID is characterized by urticaria after generalized exposure to cold air or evaporative cooling (Figure 2A, right panel), but with a negative cold stimulation test (ice cube test).[Fig Figure2B]

**Other clinical signs**

Affected patients present with vesiculobullous eruptions in the first days of life in colder areas of the body (tip of the nose, ears, and fingers). Sometimes eruptions evolve into crusted ulcerations and soft tissue destruction, which usually resolve. Some individuals may present with autoinflammatory disease, which has been labelled APLAID with no cold urticarial but blistering skin lesions upon exposure to heat or the sun, arthralgias, colitis, central nervous system and interstitial lung inflammation and autoimmunity (vitiligo, Hashimoto’s thyroiditis). Recurrent skin and sinopulmonary infections are due to B-cell deficiency. Recent work suggests that PLAID and APLAID may represent a continuum and that the term PLAID should be used to refer to all subjects with immunodeficiency (ID) caused by *PLCG2* variants [12]. 

Other PAD with dysregulated granulocyte/ mast cell function are summarized in Table 2 [8, 13, 14]. 

It has been proposed to list systemic mastocytosis also as PAD or IEI with TH2-driven manifestation as there are genetic disorders like PLAID and ADGRE2/EMR2 deficiency, in which the defect affects primarily mast cells and may play the central pathophysiological role in urticaria formation [6, 7]. We feel, however, that the clinical presentation of cutaneous and systemic mastocytosis is not that of a primarily atopic disease and therefore, mastocytosis is not included as PAD here. 

## III. Disorders affecting the adaptive immune system, defects in central tolerance induction in the thymus 

**RED FLAG: Newborn with very severe erythroderma, extensive desquamation, failure to thrive, lymphoproliferation, lymphadenopathy, and organomegaly as signs for oligoclonal T-cell expansion and failure of central tolerance.**

### SIGNATURE DISEASE: Omenn Syndrome

Hypomorphic autosomal recessive (AR)LOF variants in genes associated with severe combined immunodeficiency disorders (SCID) (*RAG1, RAG2, ZAP70, LIG4, DCLR1C, IL7RA, AK2 CARD11*, *ADA)*. Genes associated with syndromic thymic defects AR LOF variants in *PAX1* (no thymus, otofaciocervical syndrome type 2, ear abnormalities), *EXTL3* (immunoskeletal dysplasia with neurodevelopmental abnormalities; short stature; cervical spinal stenosis) or AD and AR LOF variants in *FOXN1* (recurrent, viral and bacterial respiratory tract infections; nail dystrophy) or the AD variants in *CHD7* (CHARGE syndrome), or 22q11 (Di George syndrome). 

**Pathophysiology**

In hypomorphic SCID or syndromic T-cell lymphopenia/deficiency (low T-cell receptor excision circles (TRECS)), dysregulated, highly activated, but poorly functional T cells with a highly restricted oligoclonal Vβ TCR repertoire expand massively and result in lymphadenopathy and organomegaly. The exact mechanism is unknown. In SCID, induction of central tolerance by thymic deletion may be defective (e.g., by insufficient Treg generation and clonotypic gaps in Treg specificity). The low strength of the TCR peptide MHC interaction may contribute to TH2 priming of naïve T cells and lead to eosinophilia and high levels of circulating inflammatory cytokines. Peripheral B-lymphocyte numbers are low or absent as well as levels of immunoglobulin classes other than IgE. 

**Clinical signs suggesting allergy**

The erythroderma/erythematous rash may be present at birth or evolve over the first few weeks of life (Figure 3). The initial appearances may be papular, sometimes confluent, and the skin often becomes thickened with a “leathery” texture (pachyderma). 

**Other clinical signs**

Children suffer from diarrhea, failure to thrive, and persistent uncontrolled viral infections. Hair, including the eyebrows, is often lost as the rash evolves resulting in hypo- or atrichosis (Figure 3). 

## IV. Disorders affecting the adaptive immune system, defects in peripheral tolerance induction by regulatory Tregs 

**RED FLAG: At any age, multiple, severe autoimmune diseases like enteropathy and endocrinopathies (diabetes, thyroiditis, adrenal insufficiency) as a result of failure of peripheral tolerance.**

### SIGNATURE DISEASE: Tregopathy – Immune dysregulation, polyendocrinopathy, enteropathy, X-linked (IPEX) syndrome 

hemizygous variants in *FOXP3,* X-linked recessive 

**Pathophysiology**

IPEX syndrome dramatically illustrates the non-redundant, exclusive role Tregs have in controlling human immune system homeostasis. FOXP3 deficiency is due to a disorder of transcriptional programs of different genes important for immune regulation (Figure 4). 

**Clinical signs suggesting allery**

Affected individuals may present with generalized dermatitis of unusual severity and early onset in the neonatal period) (Figure 4, right panel, above). Due to severe IgE-mediated food allergies, IPEX patients may develop acute urticaria, vomiting, or severe anaphylaxis following ingestion of dietary allergens. 

**Other clinical signs**

Children usually present in the neonatal period or during early infancy with the classic triad: Exanthema or eczema; failure to thrive (FTT) due to autoimmune enteropathy (life threatening severe diarrhea, profound dehydration and malabsorption) and autoimmune endocrinopathies (mainly neonatal type 1 diabetes or thyroiditis, adrenal insufficiency). Serious infections are common (staphylococcal, viral, fungal) facilitated by the breakdown of the barrier of the gastrointestinal tract and skin. 

Other Tregopathies are presented in Table 3 [17, 18] 

## V. Disorders affecting the adaptive immune system, defects in downstream TCR signaling 

**a. Cytoskeleton activation and immune synapse formation**

**RED FLAG: Infants, toddlers, or school-aged child with extensive, at times disfiguring, very severe cutaneous infections (e.g., viral skin infections with deep ulcerations), bleeding diathesis and autoimmune manifestations as signs for actinopathy and cytoskeletal dysfunction**

### SIGNATURE DISEASE: Arp2/3-mediated filament branching defect 

biallelic LOF variants in *ARPC1B* AR 

**Pathophysiology**

The disease is caused by defective Arp2/3 filament branching, associated with defects in the development and function of thrombocytes. ARPC1B deficiency results in the inability of T cells to extend lamellipodia upon TCR stimulation to assemble the immune synapse. Finally, it has a major impact on neutrophil motility. 

**Clinical signs suggesting allery**

Moderate-to-severe eczema is observed in more than half of the cases sometimes associated with food allergy (anaphylactic reactions) and asthma. 

**Other clinical signs**

Affected patients present early in life (mean 2 months of age, range 1 – 6 months). They suffer from eczema, infections, and recurrent or severe bleeding episodes (e.g., neonatal hemorrhagic enteritis). There is an abnormal severity of cutaneous infections (abscesses, erysipelas, extensive warts, e.g., molluscum contagiosum) (Figure 5) and respiratory tract infections as well as significant autoimmunity [19, 20]. 

Other defects in downstream TCR signaling, cytoskeleton activation, and immune synapse formation are presented in Table 4 [3, 19, 22, 23]. 

**b. CBMopathies**

AR LOF variants in *CARD11* or *MALT1*

Caspase recruitment domain (CARD) protein–B cell CLL/lymphoma 10 (BCL10)–MALT1 paracaspase (MALT1) (CBM) complexes are critical signaling adaptors. Variants that alter the function of members of the CBM complex lead to diseases called CBMopathies two of which present with atopy (Figure 7A) (Table 5). 

## VI. Disorders affecting the innate and adaptive immune system, defects in cytokine signaling 

**a. Via STAT3 pathway**

**RED FLAG: Any age, bacterial (particularly staphylococcal) and fungal infections, poor inflammatory responses, significant lung pathology together with connective tissue anomalies**

### SIGNATURE DISEASE: STAT3 deficiency synonymous to autosomal dominant hyper-IgE syndrome (AD-HIES) 

LOF dominant negative variants in *STAT3*

**Pathophysiology**

The failure of STAT3-induced RORγt and subsequently IFNγ, TH1, and TH17 induction leads to a TH2 shift, extremely elevated total serum IgE levels, and marked eosinophilia. The deficiency of TH17 cells and the neutrophil dysfunction associated with it cause a severely impaired immunity to staphylococci and fungi that cause abscesses and lung pathology. In the context of infections, lack of IL6-signaling is associated with poor upregulation of the inflammatory marker CRP, because of reduced or absent STAT3 transcriptional activity. Osteoblast-associated genes are downregulated and angiogenesis is impaired due to dysfunctional endothelial cells/VEGF3 production. 

**Clinical signs suggesting allergy**

Affected patients may have a pustular newborn rash and will develop chronic eczematoid dermatitis, peripheral eosinophilia, and eosinophilic tissue infiltration, in particular in the gastrointestinal tract. The elevated levels of serum IgE in patients with AD-HIES are probably an associated abnormality, rather than central to the pathogenesis of the disorder. 

**Clinical**

Patients suffer from recurrent staphylococcal skin abscesses. Due to the chemotaxis defect, those abscesses are deemed as cold as they lack the cardinal signs of inflammation: calor (warmth), rubor (redness), dolor (pain), and tumor (swelling). Sinopulmonary infections in combination with the connective tissue disorder lead to abnormal lung cysts (pulmonary abscesses, pneumatoceles) most often due to *S. aureus*, *Aspergillus,* *Pneumocystis jirovecii.* Connective tissue pathology involves facial dysmorphism (broad nasal bridge) (Figure 6, right upper panel), retained primary teeth (right lower panel), osteoporosis, pathologic fractures, scoliosis, hypermobile joints, arterial tortuosity, and coronary/cerebral aneurysms. Patients on oral contraceptives or during pregnancy are at an increased risk of thromboembolic events (due to the vascular abnormalities in HIES), miscarriage, and other complications. 

Other diseases with STAT3 dysfunction are listed in Table 6 [4, 5, 6, 7, 24, 25]. 

**b. Via JAK1/STAT5b, TGF-β/ERBIN, and IL4/STAT6**

PAD due to CBMopathies and cytokine signaling dysfunction are summarized in Table 6 [4, 7, 26]. 

## PART 2 

**Navigation strategy: Map your trip to the allergy ocean using red flags as landmarks and align them with the PAD compass reading (** Table 7 **) (** Figure 8 **).**

The chronologic structured and precise documentation of symptoms and signs based on history and physical examination is key. Red flags and clinical landmarks to identify PAD patients or families are presented in Table 7 and Figure 8. The statement by Peng and Kaviany [29] summarizes one part of our strategy towards finding the extremely rare PAD within millions of allergic individuals. “...no laboratory assays are able to substitute for the essentiality of a detailed clinical timeline and history” [27]. 

**Employ genetic analyses methods most efficiently**

High-throughput sequencing (HTS) or next-generation sequencing (NGS) enables rapid and cost-effective parallel analysis of many genes. It has replaced single geneSanger sequencing (“first-generation sequencing”) in routine diagnostics. Most diagnostic laboratories today offer the so-called short-read NGS technique, in which patient DNA segments (short reads) of around 150 base pairs are compared with a reference sequence of the human genome (“alignment”). While they cover large regions or even the entire genome rather precisely, all short-read-based HTS techniques have the disadvantage that, methodologically, they cannot reliably resolve certain genomic regions, e.g., repeat expansions or larger homologous genomic regions. For addressing these questions, long-read whole genome sequencing (WGS) might be suitable. Advantages and disadvantages of different HTS assays are summarized in Supplemental Table 1. 

**WGS is the best compass (gold standard) for diagnosing PAD**

Many PAD do have poorly defined phenotypes and some patients are acutely ill. The conventional approach in the past with narrow hypotheses involving analysis of single or small groups of genes is likely to be more time-consuming, less successful compared to WGS and may lead to significant misinterpretation regarding copy number variants (CNV) (deletions, duplications) and structural variants (SV) (inversions, translocations). In the past, the use of these less efficient approaches has led to a loss of precious time. WGS allows more reliable detection of CNV and SV and re-iterative data analysis at later time points. This has the potential of eventually finding the diagnosis, e.g., in a third or fourth interpretation cycle. Finally, identification of novel genes is not unlikely as it is estimated that there are many more PAD waiting to be unraveled by the help of WGS. 

**Compass readings – interpretation**

The most difficult, but most important step for clinical geneticists is to assess discrepancies between the reference and patient sequences (called variants). Variants need to be interpreted regarding their medical relevance as candidates for causing the patient’s phenotype (e.g., PAD). Genetic analysis is at great danger of over-interpretation (candidate variants identified; incorrect association with phenotype without functional proof) or under-interpretation of data (no candidate variants identified; premature exclusion of PAD). In order to designate pathogenicity to a specific variant, the American College Of Medical Genetics And Genomics (ACMG) and the Association for Molecular Pathology (AMP) developed guidance that provides a framework for sequence variant interpretation by utilizing common types of variant evidence, such as population data, computational data, functional data, and segregation data [28, 29]. Variants are classified into benign and likely benign variants (class 1 or 2; likelihood > 99.9 or > 90% of being not pathogenic), variants of unknown significance (VUS) (class 3, likelihood between 10 and 90% of being pathogenic), likely pathogenic and pathogenic variants (class 4 or 5 variants, likelihood of > 90 to 99% or > 99% to be pathogenic). Direct clinical consequences are currently recommended only for class 4 and class 5 variants. 

### WGS scenario 1: Candidate variant identified, relation to PAD phenotype unclear 

A precise description of variants regarding nomenclature, allelic distribution, and presumed function is mandatory. Variants may be germline or somatic (meaning they may be located only in selected cell populations). The allelic distribution regarding homozygosity, heterozygosity, compound heterozygosity is determined. LOF variants can result in zero function or they can be hypomorphic. Variants can be hypermorphic GOF or they can be both (multimorphic). In patients from highly consanguineous families, PAD phenotype might be the result of not only one single, but several variants. In many genetic diseases, there is highly variable expressivity or incomplete penetrance of diseases. Trio (index and parent) and segregation analysis can be helpful and improve the diagnostic yield [30]. 

The interdisciplinary assessment of the plausibility of the variant in children and adults with (suspected) PAD by physicians, clinical immunologists, pediatric dermatologists, clinical geneticists, and cell biologists in regular multidisciplinary conferences plays a great role in the interpretation of VUS. In the light of new clinical information or newly characterized class 4 and 5 variants, it is key to iterate and reiterate the analysis of the genetic data until the diagnosis is made. 

### WGS scenario 2: No candidate variant identified, but PAD phenotype 

False-negative results may stem from using inadequate material, inaccuracies of the sequencing method, and host-related factors. Somatic variants can occur in certain cell populations only (e.g., GOF *STAT5B* variants in eosinophils) and may not be detected by HTS until cell populations are sorted. Regarding inaccuracies, in target gene panels (TGP) or whole exome sequencing (WES), CNV/SV are particularly at risk of being missed (e.g., single exon deletions in *DOCK8* or genes with homologous regions). 

Factors in the host may be

- Variant not yet associated with a PAD (potentially novel PAD)
- Reversion of variants (e.g., SCID revertants of *RAG1* variants to wild-type)
- Host phenotype phenocopy of PAD (e.g., caused by autoantibodies or epigenetic alterations)

A negative genetic analysis in the presence of a significant phenotype should be re-evaluated and analyses of the genetic data reiterated from time to time, as there may be new clinical information (e.g., peculiar opportunistic infection), a new PAD, or a new methodology at hand. Again, this can only be done by a multidisciplinary approach with regular meetings (see above). 

## Outlook – sailing to new horizons 

Long-read WGS (third-generation sequencing, read length of several thousand bases) may be suitable and enter routine diagnostics soon. This in turn will lead to a further increase in the diagnostic yield. Conventional physical examination by the physician will soon be aided by next genertion phenotyping (NGP) using artificial intelligence (AI) like the GestaltMatcher data base (GMDB). In pilot studies, GMDB has been shown to diagnose syndromic IEI in a reliable manner (unpublished data). However, analyzing and predicting the effect of a VUS on the protein level still remains a challenge. The functional validation of VUS will be sped up by data sharing on platforms (like GeneMatcher) and other publicly accessible online resources and the use of AI. Multiomics (epigenome, transcriptome, microbiome, proteome, etc.) will aid genomics and most importantly help to functionally confirm or exclude variants with regard to a PAD very rapidly, which up to now is still extremely time-consuming or not realistic. We will likely detect PAD much earlier which will be a benefit for PAD patients and their families. 

## Authors’ contributions 

TN: conceptualization, methodology, writing – original draft. EV: methodology, writing – review and editing, visualization, proof reading. SVH: conceptualization, writing – review and editing, proof reading. 

## Acknowledgment 

We thank Andrea Groth (HKK) for invaluable help in the preparation of the manuscript. 

## Funding 

EV is supported by a grant of the Elternverein für krebskranke Kinder Krefeld, Germany. TN: Funding of clinical research (KIDSSAFE study) by the German Joint Federal Committee (Gemeinsamer Bundesausschuss (G-BA) composed of sectors of the German healthcare system (Health Insurance Funds, Healthcare Providers: Patient Representatives); EVC; Funding from the Fanconi Cancer Foundation, the Deutsche Fanconi-Anämie-Hilfe e.V., Deutsche Forschungsgemeinschaft (DFG) project number 530200248 and the Elternverein Kinderkrebshilfe Krefeld and royalties from a publication relating to health education. 

## Conflict of interest 

TN: Non-profit organizations (travel expenses, no fees): Jeffrey Modell Foundation (parent organization, donations from the industry); info4pi.org; PENTA Global Pediatric Research Network (with donations from industry); penta-id.org; JIR Cohort (research network with donations from industry); Publishers (royalties): UpToDate, Inc; Springer, Elsevier; Indirect interests (membership) Transparency International; Parents’ associations (children with cancer Krefeld, children’s cancer clinic Düsseldorf); SvH: None. 

**Figure 2A.**
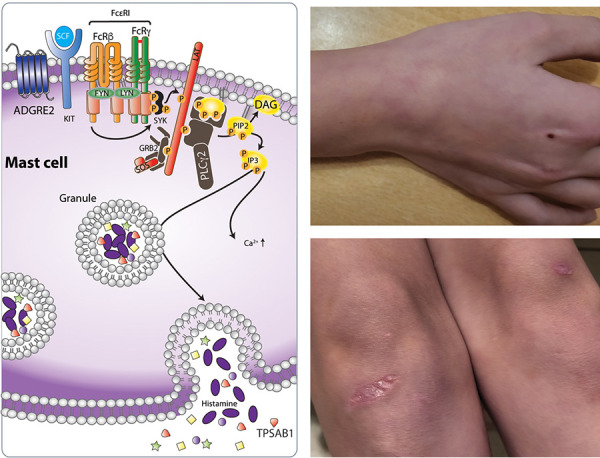
Physiology of mast cell activation (left panel) and clinical images of an 8-year-old girl with PLCγ2-associated antibody deficiency and immune dysregulation (right panel; PLCG2 variant c.313G>A p.(Val10Ile), Department of Human Genetics, University of Göttingen) and urticaria on the right wrist after exposure to cooling (swimming pool) (above) and vesiculobullous eruptions on colder areas, knees (below). SCF is the primary growth factor for mast cells, KIT, the cognate receptor for SCF. EGF-like module-containing mucin-like hormone receptor-like 2 (EMR2) is encoded by *ADGRE2*. Attachment to the membrane is mediated by a noncovalently bound subunit of the receptor, which activates EMR2/ADGRE2 in mast cells, when forcefully dissociated by physical shearing forces/mechanical stress (vibration), leading to degranulation. Phosphoinositide-specific phospholipase Cγ2 (PLCγ2) is important for B-cell differentiation and function. In mast cells, PLCγ2 is downstream of the IgE receptor and catalyzes the hydrolysis of phosphatidylinositol 4, 5-bisphosphate to the secondary messengers inositol triphosphate (IP3) and diacylglycerol (DAG). IP3 induces the release of Ca2+ from the ER. PLCγ2 can be activated by cold temperature, which leads to spontaneous calcium flux and degranulation. Tryptase (TPSAB1) is present in mast cell secretory granules. α- and β-tryptases form tetramers stabilized by heparin and are released after degranulation, where they contribute to allergic inflammation, inducing pruritus (adapted from Carlberg and Velleuer. Molecular Immunology: How Science Works, Publisher, Springer International Publishing, 2022 and [6, 7, 8]).

**Figure 2B. Figure2B:**
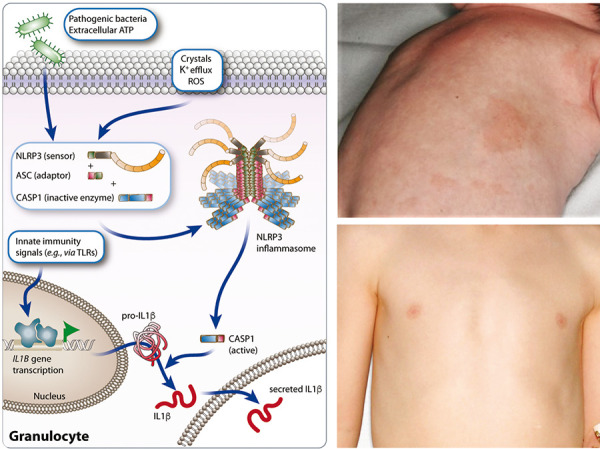
Physiology of granulocyte NLRP3 inflammasome activation (left panel) and a 2-month-old boy with CINCA/NOMID (right panel; GOF variant in *NLRP3*; Institute for Clinical Chemistry, Ludwig Maximilian University, Munich, Germany) with continuous fever since birth, urticarial rash in changing locations (above), marked eosinophilia (up to 35,000/μL) and fast recovery after institution of IL1-blocking treatment (Anakinra) (below). The NLRP3 inflammasome is a multiprotein complex composed of NLRP3, adapter protein ASC, and protease caspase-1. Upon various stimuli (e.g., microbial ligands, crystals) NLRP3 inflammasome activates caspase‐1 to induce the release of proinflammatory IL1β (adapted from Carlberg and Velleuer. Molecular Immunology: How Science Works, Publisher, Springer International Publishing, 2022).

**Table 1.** Other primary atopic diseases with disrupted epithelial and/or mucosal skin barrier [9, 10, 11].

Disease	**OMIM-P**	**Gene**	**Inheritance**	**Phenotype**
Filaggrin deficiency	#146700	*FLG*	AD or AR (LOF)	– Ichthyosis vulgaris – Early-onset persistent atopic eczema – Elevated risk of food allergy and eczema herpeticum
Severe dermatitis – multiple allergies – metabolic wasting syndrome (SAM)	#615508	*DSG1; DSP*	AD or AR (LOF)	– Life-threatening condition, severe dermatitis, multiple allergies, metabolic wasting – Hypotrichosis, palmoplantar hyperkeratosis, enamel defects, recurrent (skin) infections – In some patients little systemic involvement
Peeling skin syndrome type B	#270300	*CDSN*	AR (LOF)	– Lifelong patchy peeling of the skin with chronic pruritus – Frequent food allergy, recurrent skin infections – Usually no failure to thrive
CARD14 deficiency	#602723	*CARD14*	AD (LOF)	– Severe atopy, severe pyogenic and viral skin and respiratory tract infections due to impaired NFkB activation and impaired epidermal secretion of antimicrobial peptides

AD = autosomal dominant; AR = autosomal recessive; LOF = loss of function.

**Table 2.** Other primary atopic disorders with dysregulated granulocyte / mast cell function [8, 13, 14].

**Disease**	**OMIM-P**	**Gene**	**Inheritance**	**Phenotype**
**Cryopyrin-associated periodic syndromes (CAPS)** composed of 1. Muckle-Wells syndrome 2. Familial cold autoinflammatory syndrome (FCAS1) 3. Neonatal onset multisystem inflammatory disease (NOMID) or chronic infantile neurologic cutaneous and articular syndrome (CINCA)	#191900 #607115 #120100	*NLRP3*	AD (GOF)	– Maculopapular, non-pruritic and most predominantly urticarial rashes – Arthritis, chills, fever and leukocytosis, e.g., after cold exposure (FCAS1) – Recurrent fever, musculoskeletal symptoms, abdominal and thoracic serositis, headache, ophthalmic and auditory nerve inflammation potentially leading to deafness and blindness preventable by IL1-directed treatment – Severity of symptoms variable between and within conditions, and not indicative of a particular disease
***NLRC4* or *NLRP12*-associated autoinflammatory diseases**	#616115 #611762	*NLRC4 * *NLRP12*	AD (GOF)	Fever, arthritis/arthralgia, rash, abdominal pain, diarrhea, myalgia/fatigue and conjunctivitis triggered by cold exposure

AD = autosomal dominant; GOF = gain of function.

**Figure 3.**
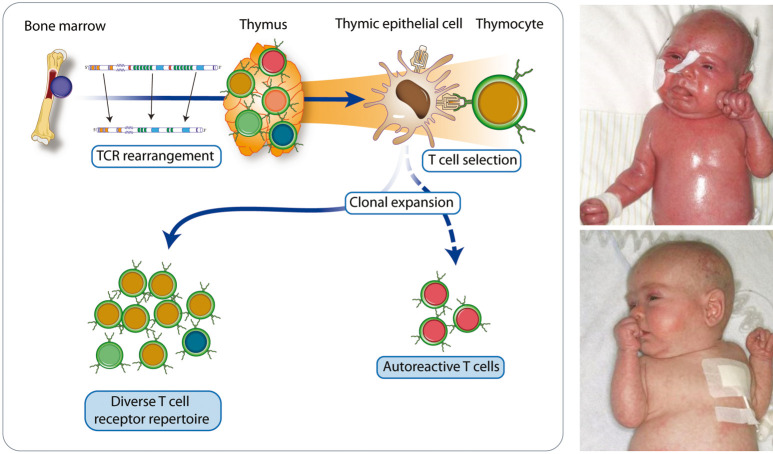
Central tolerance induction physiology (left panel) and neonate with Omenn syndrome and erythroderma (right panel, above) who was successfully treated by cord blood transplantation (below); (pictures kindly provided by H. Ott, Hannover, published in [16]). The thymic selection of T cells during the first decade of life produces millions of antigenically distinct T cells carrying a very diverse T-cell receptor (TCR) repertoire prepared to encounter millions of variants of microbial and other antigens. Some autoreactive T-cell clones are also generated. This selection process is called central tolerance induction.

**Figure 4.**
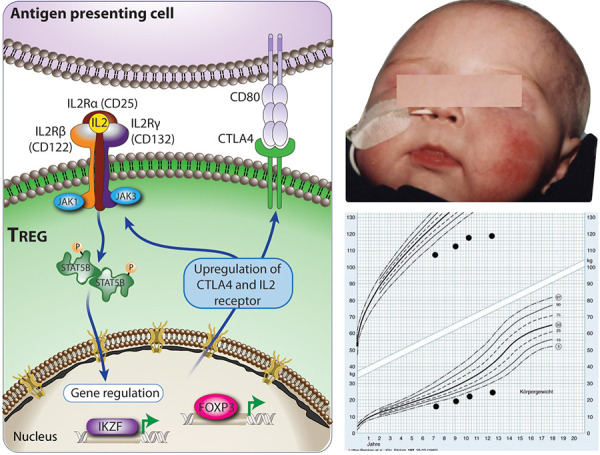
Physiology of Tregs (regulatory T cells) (left panel) and an infant with IPEX syndrome (immune dysregulation, polyendocrinopathy, enteropathy, X-linked) (right panel, above). The patient presented with severe autoimmune enteropathy and growth charts showing failure to thrive, length and weight < 3^rd^ percentile (right panel, below). Tregs induce peripheral tolerance, preventing development of autoimmune and allergic diseases. Their expression of the high affinity trimeric α/β/γ IL2 receptor (IL2R) helps them to selectively bind IL2 and draw it away from T effector cells (Teff) expressing only the low moderate affinity (β/γ) IL2R. FOXP3 is the lineage defining transcription factor for the development of Tregs. High levels of FOXP3 expression are directly linked to the suppressive capacity of Tregs. FOXP3 expression leads to the upregulation of the high affinity IL2 receptor as well as CTLA-4 on Tregs. CTLA-4 competes with the co-stimulatory molecule, CD28, for binding to CD80 and CD86 on antigen presenting cells (APCs). This is one other mechanism by which Tregs suppress immune responses. IKZF (IKAROS family zinc finger) have been shown to be critical for Treg and hematopoietic development.

**Table 3.** Other primary atopic diseases Tregopathies [17, 18].

**Disease**	**OMIM-P**	**Gene**	**Inheritance**	**Phenotype**
**Disorders of Treg transcriptional programs**				
**IKAROS-associated (IKZF1) disease**	#616873	*IKZF1*	AD (GOF or LOF)	– Onset > 1 to up to 40 years of age – Many present with atopic diseases – Autoimmunity/immune dysregulation can be profound: e.g. autoimmune cytopenias (Evans Syndrome), lymphoproliferation, plasma cell expansion (IgG4+), type 1 diabetes, thyroiditis alopecia, vitiligo, celiac disease, colitis) – Sinopulmonary infections – Expressivity of GOF variants is variable, asymptomatic individuals are common
**Disorders of IL2 or CTLA4 signaling**				
**Interleukin 2 receptor a (IL2RA (CD25)) or interleukin-2 receptor β (IL2RB (CD122))**	#606367 #618495	*IL2RA (CD25), IL2RB (CD122)*	AR (LOF)	IPEX-like (see above) and SCID-like presentations
**CTLA4 haploinsufficiency**	#152700	*CTLA4*	AD (LOF)	Onset throughout childhood and adulthood. – Lymphoproliferation, lymphoma, gastric cancer, polyautoimmunity (e.g., autoimmune enteropathies, cytopenias, skin disease, interstitial lung disease, and neurologic manifestations). – Increased severity of infections (e.g., CMV, EBV). – Often initially diagnosed as CVID – Some parents and relatives with pathogenic variants asymptomatic.

AD = autosomal dominant; AR = autosomal recessive; IPEX = immune dysregulation, polyendocrinopathy, enteropathy, X-linked; SCID = severe combined immunodeficieny; CMV = cytomegalovirus; EBV = Ebstein-Barr virus; CVID = common variable immunodeficiency.

**Table 4.** Other defects in downstream TCR signalling, cytoskeleton activation and immune synapse formation [3, 19, 21, 22].

**Disease**	**OMIM**	**Gene**	**Inheritance**	**Phenotype**
**Wiskott-Aldrich syndrome**	#301000	*WAS*	XL (LOF)	Onset: early childhood with the classic triad: – Thrombocytopenia (pathognomonic small mean platelet volume (< 5 fL)) – Recurrent severe infections (impetigo, cellulitis, skin abscesses, molluscum contagiosum; Herpesviruses, including herpes simplex and varicella-zoster virus, Epstein-Barr virus) – Eczema, often unresponsive to conventional treatment. – Early death may result from bleeding. – Autoimmunity and malignancy more common with increasing age. – Milder phenotype: X-linked thrombocytopenia without eczema or immunodeficiency.
**WIP deficiency**	#614493	*WIPF1*	AR (LOF)	– WAS-like presentation, but very early onset of a severe immunodeficiency
**NCKAP1L deficiency **(HEM-1 hematopoietic protein 1)	#618982	*NCKAP1L*	AR (LOF)	– Atopic disease and hyperinflammation, chronic hepatosplenomegaly, lymphadenopathy – Recurrent fever and upper respiratory tract infections, skin rashes, abscesses, ulcers, autoimmune manifestations and FTT
**DOCK8 deficiency**	#243700	*DOCK8*	AR (LOF)	– Extensive, disfiguring, concurrently occurring cutaneous viral infections, particularly HSV, human papillomavirus, molluscum contagiosum and varicella-zoster virus. – Invasive infections (wide spectrum of Gram-positive and Gram-negative bacteria, viruses and intracellular fungi, e.g., histoplasma capsulatum). – Mucocutaneous candidiasis and recurrent gastro-intestinal tract infections are common. – Severe and extensive food allergies. – High risk of developing malignancies, particularly lymphomas and squamous cell carcinomas.
**CARMIL2 (RLTPR) deficiency**	#618131	*CARMIL2*	AR (LOF)	– Onset early infancy; – Atopic and seborrheic dermatitis and psoriasis-like rashes. – Viral (EBV, CMV, and varicella), bacterial, mycobacterial and fungal infections – Early or very early onset inflammatory bowel disease (VEOIBD) – Autoimmune polyendocrinopathy syndrome (APS) – Characteristic feature is EBV-associated leiomyoma (<20% of cases)
**STK4 deficiency**	#614868	*STK4*	AR (LOF)	– Onset at school age – Infections (cutaneous viral infections, recurrent pneumonia, EBV-associated lymphoproliferation) – Autoimmune or inflammatory diseases and atopic dermatitis/atopy
**TBX21 deficiency ** **(T-bet, T-box transcription factor 21) **	#619630	*TBX21*	AR (LOF)	– Mendelian susceptibility to mycobacterial disease (MSMD) – Persistent upper airway inflammation
**Moesin-associated immunodeficiency (X-MAID)**	#300988	*MSN*	XLR (LOF)	– Skin manifestations, mainly eczema, molluscum contagiosum and increased susceptibility to bacterial and viral infections and VEOIBD

XL = X linked; LOF = loss of function; AD = autosomal dominant; AR = autosomal recessive; XLR = X-linked recessive; WAS = Wiscott-Aldrich syndrome; FTT = failure to thrive; HSV = Herpes simplex virus; EBV = Ebstein-Barr virus; CMV = cytomegalovirus; VEOIBD = very early onset inflammatory bowel disease.

**Figure 5.**
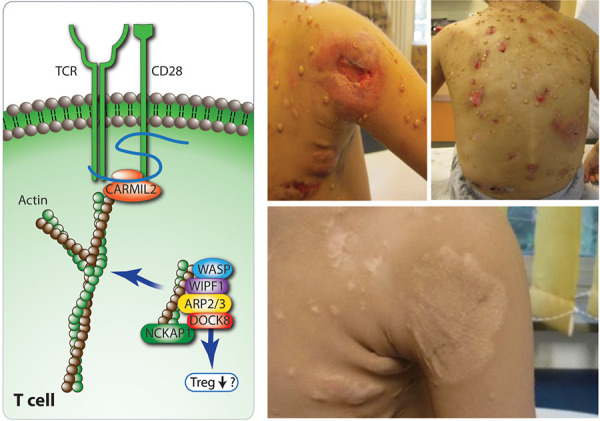
Physiology of the actin cytoskeleton (left panel) and a 3-year-old boy (right panel, above) with ARPC1B (actin related protein 2/3 complex subunit 1B) deficiency, presenting with ulcerating severe cutaneous viral infections (left shoulder and on the back) and after successful stem cell transplantation at 5 years of age (right panel, below). Rearrangement of actin cytoskeletons is key for immune cell activation, migration and adhesion. Human actin-related protein 2/3 complex (Arp 2/3) has ARPC1 component isoforms (ARPC1B, expressed in blood cells, ARPC1A in non-hematopoietic tissues). WASP (Wiskott Aldrich syndrome protein), WIPF1 (WASP interacting protein family member 1), DOCK8 (dedicator of cytokinesis 8), and NCKAP1 (Nck-associated protein 1 also called HEM1 (hematopoietic protein 1)). The RLTPR or CARMIL-2 protein, the TCR, and CD28 form microclusters that interact with actin and are key for the formation of the immunological synapse. Moesin (MSN) links actin filaments to the plasma membrane which increases cell rigidity and polarity and STK4 controls the translocation of vesicles to the surface and can activate actin (both not shown in Figure).

**Figure 6.**
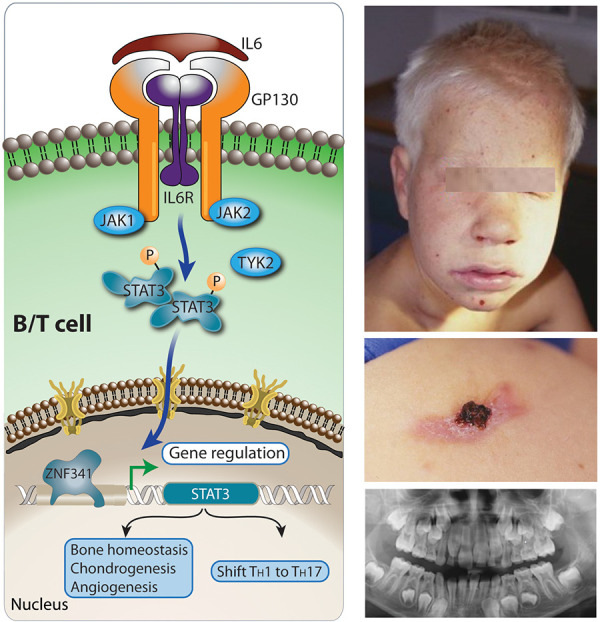
Physiology of STAT3 signaling (left panel) and a 7-year-old boy with coarse facial features (right panel, above), eczema, cold skin abscesses (middle) and delayed dentition at 10 years of age (below, different patient). IL6 signals through the IL6RST (signal transducer of IL6 receptor complex), composed of glycoprotein 130 (GP130) and the IL6R. Other cytokines of the IL6 cytokine family utilize GP130 encoded by *IL6ST* (IL11, OSM, LIF, CNTF, CT-1, CLCF1, IL27, IL35, and IL39). IL6 then activates through JAK-STAT signal transduction the zinc finger protein 341 (ZNF341) TF, which promotes STAT3 transcription. IL6 and IL23 activate *STAT3*, which turns on *RORC*, encoding RORγt the master regulator for a TH1/TH17 T-cell response shift as well as chondrogenesis, bone repair, angiogenesis/vascularization (induction of vascular endothelial factor 3 (VEGF3)) [23]. The phosphoglucomutase 3 (PGM3) (not shown in figure) enzyme catalyzing the isomerization of N-acetyl-glucosamine-6-phosphate to N-acetyl-glucosamine-1-phosphate during the generation of UDP-N-acetyl-glucosamine UDP-GlcNAc. During glycosylation, sugar chains are added to either proteins or lipids, using basic sugar building blocks (UDP-GlcNAcs) to make N-glycans, O-glycans, proteoglycans, and glycosylphosphatidylinositol (GPI)-anchored proteins all participating in cell signaling.

**Table 5.** PAD due to CBMopathies and cytokine signaling dysfunction [4, 7, 26].

Disease	OMIM	Gene	Inheritance	Phenotype
**CBMopathies**				
**CARD11-associated atopy with dominant interference of the NFkB signaling (CADINS)**	#617638	*CARD11*	AD (LOF)	– Very severe atopic dermatitis (close to 90% of cases) later followed by asthma and food allergy. Cutaneous viral and respiratory tract infections, potentially severe and causing FTT (e.g., due to diarrhea). Some patients display skeletal features as in STAT3 deficiency (broad nose, retained teeth).
**MALT1 deficiency**	#615468	*MALT1*	AR (LOF)	– Left untreated it is thought to be fatal – Recurrent severe bacterial viral and fungal infections of the skin and/or respiratory and gastrointestinal tract infections, FTT – Periodontal disease, aphthous ulcers, cheileitis and gingivitis.
**TGFβ pathway**				
**Transforming growth factor β receptor 1 and 2 (TGFBR 1 and 2) deficiency (Loeys-Dietz syndrome 1 and 2 )**	#609192 #610168	*TGFBR1, TGFBR2*	AD (LOF)	– IgE-mediated food allergy, EGID, allergic asthma, and atopic dermatitis. Additional connective tissue abnormalities overlapping with those seen in *STAT3* pathway disorders (hyperextensible joints, scoliosis, etc.)
**Erbb2-interacting protein (ERBIN) deficiency**	None	*ERBIN*	AD (LOF)	– Significant EGID, connective tissue abnormalities. – Enhanced TGF-β pathway activation leading to increased IL4Rα expression and enhanced Th2 differentiation and IgE production
**JAK1/STAT5b pathway**				
**JAK1 GOF**	#618999	*JAK1*	AD (GOF)	– Severe atopic dermatitis and allergic asthma. Immune dysregulation such as autoimmunity (e.g., thyroid disease), poor growth, hepatosplenomegaly, eosinophilic enteritis.
**STAT5B hypereosinophilic syndrome**	#102578	*STAT5B*	Somatic (GOF) in multiple hematopoietic lineages	– Neonatal onset dermatitis, urticarial rash and diarrhea, neonatal hypereosinophilia. – Patients with somatic GOF STAT5B variants have presented with leukemia and lymphomas.
**STAT5B deficiency**	#618985	*STAT5B*	AD (dominant negative) and AR (LOF)	– Recurrent viral infections due to poor IL-2-mediated effector functions and chronic pulmonary disease (lymphocytic interstitial pneumonitis). – Extreme short stature due to the roles of STAT5b in growth factor signaling (Growth-hormone insensitive dwarfism, dysmorphic features). – Atopy, e.g., eczema. Prominent autoimmunity similar to IPEX (Modestly reduced Treg number and function). The AD form of STAT5B deficiency has growth-failure, eczema, but no immunodeficiency.
**IL4/STAT6 pathway**				
**STAT6-GOF disease (signal transducer and activator of transcription 6)**	#620532	*STAT6*	AD (GOF)	– Severe early-onset, multi-system allergic disease, e.g. severe and treatment-resistant dermatitis, marked eosinophilic gastrointestinal disease. – Skin and respiratory infections as well as lymphadenopathy, cobblestone-like appearance of the buccal mucosa, polypoid nodules in the intestinal tract, and notably, B-cell lymphomas. – Non-immunological features: some resembling AD HIES, some are additional: renal fibrosis, short stature and hypotrichosis

GOF = gain of function; LOF = loss of function; AD = autosomal dominant; AR = autosomal recessive; FTT = failure to thrive; EGID = eosinophilic gastrointestinal disease; IPEX = immune dysregulation, polyendocrinopathy, enteropathy, X-linked; HIES = hyper IgE syndrome .

**Table 6.** Other primary atopic disorders with STAT3 dysfunction [4, 5, 6, 7, 24, 25].

**Disease**	**OMIM**	**Gene**	**Inheritance**	**Phenotype**
**ZNF341 deficiency**	#618282	*ZNF341*	AR (LOF)	– Phenocopy of AD-HIES
**Partial IL6 signal transducer (IL6ST) (GP130 deficiency)**	#619752	*IL6ST*	AD (LOF)	– Demonstrates phenotypic overlap with AD HIES but also diarrhea, keratitis, and neurodevelopmental delay.
**Complete IL6 signal transducer (IL6ST) (GP130 deficiency) **	#618523	*IL6ST*	AR (LOF)	– Death in utero or in neonatal period in most affected individuals. – Stuve-Wiedemann-like syndrome; skeletal dysplasia, osteoporosis, lung dysfunction, renal abnormalities, thrombocytopenia, eczema.
**IL6R deficiency**	#618944	*IL6R*	AR (LOF)	– Atopic dermatitis, elevated IgE, bacterial sinopulmonary infection, and substantial skin and soft tissue infections – often due to staphylococcus. No connective tissue abnormalities
**Phosphoglucomutase 3 deficiency**	#615816	*PGM3*	AR (LOF) hypomorphic	– Syndromic immunodeficiency due to glycosylation defect. Atopic dermatitis, bronchiectasis, and scoliosis but lack of characteristic AD HIES facies, chemotaxis defects, cold abscesses and connective tissue disease. – Developmental delay, primary neurocognitive deficits, hypomyelination and skeletal dysplasia

AD = autosomal dominant; AR = autosomal recessive; HIES = hyper IgE syndrome.

**Figure 7.**
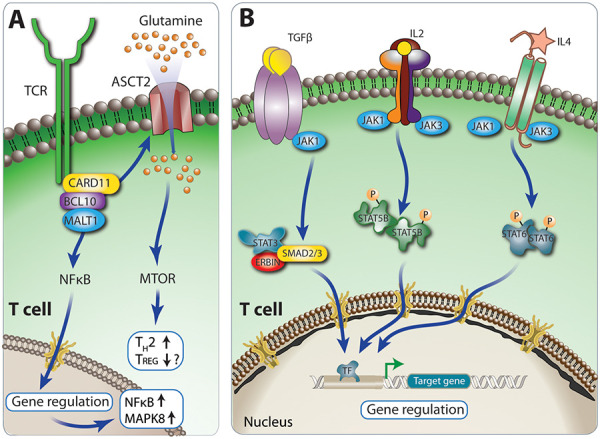
A: CBM physiology CBM complexes are critical TCR signaling adapters. Antigen receptor stimulation leads to phosphorylation of CARD 11. Activated CARD 11 is recruited into the CBM complex to the ubiquitin regulatory proteins LUBAC and TRAF6 which ligate unique ubiquitin chains to BCL 10/MALT1 (not shown in Figure) and eventually lead to the activation of NFkB, JNK and ASCT2 regulating glutamine metabolism and activating MTOR. B: JAK1/STAT5/ TGFβ/STAT6 Physiology. TGFβ has roles in immune tolerance, cell cycle arrest, and wound healing. TGFβ signals through TGFBR1 and 2 Receptor. TGFβ increases IL4Rα expression and leads to T helper type 2 (TH2) differentiation and IgE production. ERBIN is a negative regulator of transforming growth factor (TGF)-signaling through interaction with SMAD proteins. STAT3 induces and then forms a complex with ERBIN. STAT5 is activated by signaling through receptors for various cytokines including IL2, hematopoietic growth factors, as well as growth hormone. STAT5B is an important regulator of FOXP3 expression, the essential TF for Tregs. STAT6 signaling induces activation of group 2 innate lymphoid cells (ILC2s), differentiation of TH2 cells from naïve CD4+ T cells and induction of T follicular helper (TFH) cells, which are crucial for B-cell affinity maturation and immunoglobulin class-switch recombination (CSR) in the germinal center. In non-immune cells, IL4/IL13 STAT6 is involved in collagen production of fibroblasts, the development of mucus-secreting goblet cells, eosinophil-attracting chemokine production from epithelial cells and induction of bronchial smooth muscle hyper-responsiveness.

**Table 7.** Screening landmarks F, AD, ID, SY regarding history and physical findings. Presence of Red flags in > 2 landmarks will trigger whole genome analysis. Abnormal findings in lab investigations are only significant in presence of landmarks F, A, ID, SY.

**Land-mark**	**Category**	**Tools**	**Red Flag**
F	**Family history**	Draw a pedigree^§^, find inheritance pattern; know geographical distributions of consanguinity^$^	– Presence of family members with IEI; family members fulfill other landmarks (see below)
A	**Atopy disease history **	Get details: onset, extent, course	– Onset: at birth/first 1 – 2 months of life – Extent: multiple simultaneous atopic manifestations such as severe asthma, life-threatening anaphylaxis, allergic rhinoconjunctivitis, multiple food allergies, eosinophilic esophagitis and gastroenteritis, protein-losing enteropathy – Course: very severe, not responsive to standard therapy
ID	**Immunodeficiency, non-atopic past medical history**	Evaluate pathological susceptibility to infections, signs of immunodysregulation, past medical history (ELVIS**, GARFIELD,** see next column)	General – Failure to thrive^+^ – Malignancy (e.g., lymphomas, leiomyosarcoma) Pathological susceptibility to infections (ELVIS)** – Unusual, opportunistic pathogens: e.g. pneumocystis – Localization: unusual, e.g., organ abscesses – Course: exceptionally long duration – Intensity: exceptionally severe, e.g., admission to intensive care unit. – Sum: Too many, e.g., in adults, >3 or in children > 6 infections per year which require treatment (including antibiotics) and each lasting more than 4 weeks Immunodysregulation (GARFIELD)** – Granuloma – Autoimmune disease – Recurring fever and chronic inflammation – Unusual eczema – Lymphoproliferation – Chronic, inflammatory bowel Disease
SY	**Syndromic disease**	Skilled examination by experienced physician, clinical geneticist; supplementary use of artificial intelligence (next generation phenotyping (e.g., GestaltMatcher)^++^	Key clinical findings such as hypotrichosis, neurodevelopmental delay, skeletal or connective tissue abnormalities (e.g., coarse facies, vascular anomalies, bleeding)
**! Caveat !:** Except newborn SCID screening, results of conventional laboratory studies are not sufficient to make a definitive diagnosis of PAD. High levels of IgE or eosinophilia do occur in many PADs but are also common in non-primary allergic diseases. Abnormal findings in laboratory investigation count only as significant in the presence of landmarks F, A, ID, SY.			
LI	**Lab investigations**	SCID newborn screening; full blood count; serum IgG, IgA, IgM, IgE; specific IgE panels to common aeroallergens and food allergens; Skin prick tests; urine glycans, serum tryptase; N- und O-glycosylation in serum transferrin electrophoresis, flow cytometry with immunodeficiency adapted markers; mast cell metabolites (e.g., histamine, leukotriens) in urine	– Pathological TREC screening – Abnormal absolute numbers and relative percentages in full blood count (e.g., cytopenia; neutropenia, lymphopenia, thrombocytopenia, anemia) – Low IgG- SD age-adjusted; IgA < 5 mg/dL – Very high TH2 biomarkers: severe eosinophilia (>5,000 cells/mm^3^); serum total IgE > 2,000 kU

IEI = inborn errors of immunity; SCID = severe combined immunodeficiency; PAD = primary atopic disease; TREC = T cell receptor excision circle;SD = standard deviation.^&^Online resources and instructions on How to Draw a Pedigree - Iowa Institute of Human Genetics (uiowa.edu); (Definition of pedigree - NCI Dictionary of Genetics Terms - NCI (cancer.gov); ^$^Consang.net. **acronyms adapted from the German guideline for Diagnostics in Primary immunodeficiencies (S. Farmand, manuscript in preparation) and [31]; ELVIS = Erreger (pathogens), Lokalisation (localization), Verlauf (course), Intensität (intensity), Summe (sum); GARFIELD = Granulome (granuloma), Autoimmunität (autoimmunity), Rezidivierendes Fieber (recurrent fever), Darmentzündung (chronic inflammatory bowel disease). ^+^Growth hormone evaluation and bone age study if there is short stature; delayed bone age and growth hormone deficiency may be found. ^++^www.db.gestatmatcher.org.

**Figure 8:**
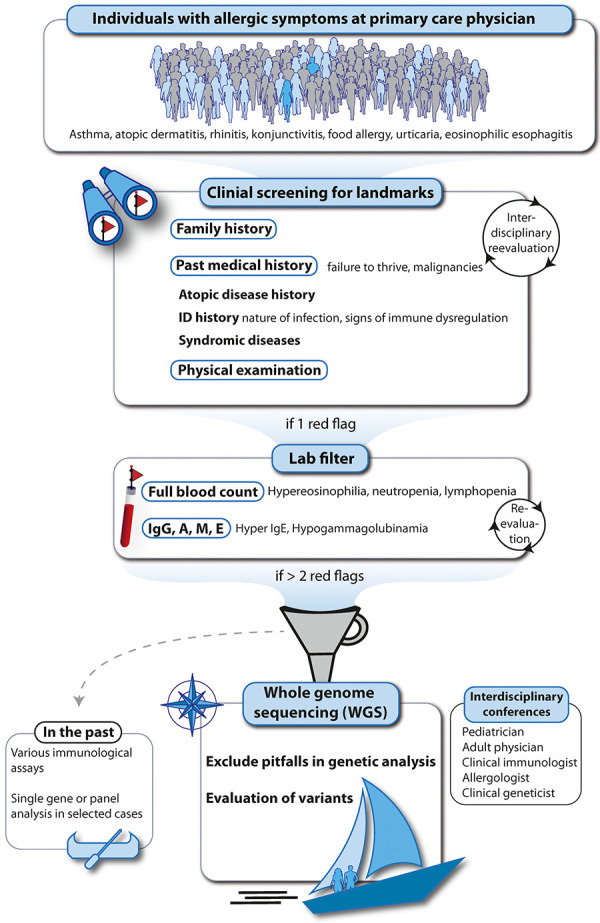
Rational, clinical landmark-based approach to identify patients/families with primary atopic disorders by early, upfront whole genome sequencing. If > 2 red flags are fulfilled, genetic analysis is indicated. In the following sections we discuss the different methods of genetic analysis and the cornerstones of genetic result interpretation in individuals with (suspected) primary atopic disorder. Details on red flags are given in Table 7.
